# Health Benefits of Montmorency Tart Cherry Juice Supplementation in Adults with Mild to Moderate Ulcerative Colitis; A Placebo Randomized Controlled Trial

**DOI:** 10.3390/life15020306

**Published:** 2025-02-17

**Authors:** Jonathan Sinclair, Graham McLaughlin, Robert Allan, Johanne Brooks-Warburton, Charlotte Lawson, Shan Goh, Terun Desai, Lindsay Bottoms

**Affiliations:** 1 Research Centre for Applied Sport, Physical Activity and Performance, School of Health Social Work & Sport, University of Central Lancashire, Preston PR1 2HE, UK; 2 School of Life and Medical Sciences, University of Hertfordshire, Hatfield AL10 9AB, UK; 3 Gastroenterology Department, Lister Hospital, Stevenage SG1 4AB, UK; 4 School of Pharmacy and Biomedical Sciences, University of Central Lancashire, Preston PR1 2HE, UK; 5 Department of Clinical, Pharmaceutical and Biological Science, School of Life and Medical Sciences, University of Hertfordshire, Hatfield AL10 9AB, UK; 6 Division of Surgery & Interventional Science, University College London, London WC1E 6BT, UK

**Keywords:** ulcerative colitis, inflammatory bowel disease, Montmorency tart cherry, complementary medicine, randomised controlled trial

## Abstract

Aims: Ulcerative colitis (UC) significantly impacts individuals’ self-perception, body image, and overall quality of life, while also imposing considerable economic costs. These challenges highlight the necessity for complementary therapeutic strategies with reduced adverse effects to support conventional pharmacological treatments. Among natural interventions, Montmorency tart cherries, noted for their high anthocyanin content have emerged as a natural anti-inflammatory agent for UC. The current trial aimed to investigate the effects of Montmorency tart cherries compared to placebo in patients with mild to moderate UC. Materials and methods: Thirty-five patients with UC were randomly assigned to receive either placebo or Montmorency tart cherry juice, of which they drank 60 mL per day for 6 weeks. The primary outcomes and health-related quality of life, measured via the Inflammatory Bowel Disease Quality of Life Questionnaire (IBDQ), and the secondary measures, including other health-related questionnaires, blood biomarkers, and faecal samples, were measured before and after the intervention. Linear mixed-effects models were adopted to contrast the changes from baseline to 6 weeks between trial arms. Effect sizes were calculated using Cohen’s *d*. Results: There were significantly greater improvements in the IBDQ (22.61 (95% CI = 5.24 to 39.99) *d* = 0.90) and simple clinical colitis activity index (−3.98 (95% CI = −6.69 to –1.28) *d* = −1.01) in the tart cherry trial arm compared to placebo. In addition, reductions in faecal calprotectin levels were significantly greater in the tart cherry trial arm compared to placebo (−136.17 µg/g (95% CI = −258.06 to –4.28) *d* = −1.14). Loss to follow-up (N = 1) and adverse events (N = 1) were low and compliance was very high in the tart cherry (95.8%) trial arm. Conclusions: Given the profoundly negative effects of UC on health-related quality of life and its fiscal implications for global healthcare systems, this trial indicates that twice-daily tart cherry supplementation can improve IBD-related quality of life as well as the severity of symptoms and therefore may be important in the management of UC.

## 1. Introduction

Ulcerative colitis (UC), a form of inflammatory bowel disease (IBD), is defined by persistent, non-infectious inflammation of the intestinal mucosa, marked by alternating phases of flare-ups and remissions [1]. In the United States, the prevalence of UC is notably high, reaching up to 500 cases per 100,000 people [2]. Comparable incidence rates have been reported in the United Kingdom [3], with the prevalence of UC steadily increasing across numerous developed countries [4]. This increasing prevalence, coupled with the significant morbidity and economic burden associated with UC, poses a substantial clinical challenge. The average direct cost of managing UC has been estimated to exceed USD 3500 per patient annually, with a large portion of these costs attributed to medication expenses [5]. For instance, a single drug used to treat UC in the UK incurs an annual cost of GBP 400 million to the health service [6]. Furthermore, recent health economic studies have identified that the pharmaceutical treatments for UC rank among the most expensive within the U.S. healthcare system [7]. As a result, UC necessitates increased financial investment and infrastructure to ensure effective long-term disease management [4]. In the United Kingdom alone, the direct financial impact of UC exceeds GBP 720 million per year, with the prevalence in Europe rising by approximately 0.3% annually [8,9].

UC habitually presents in early adulthood, with its chronic and relapsing course exerting a substantial influence on patients’ health perceptions, self-image, and overall quality of life [10]. This impact is particularly severe during active disease phases, where individuals report markedly reduced quality of life compared to those in remission [10]. Additionally, UC is often associated with increased psychological distress, heightened anxiety, and an amplified sensitivity to bodily sensations. Additionally, patients tend to perceive lower levels of social support in comparison to healthy individuals [11]. Various factors contribute to the reduced health-related quality of life observed in individuals with UC, including limited engagement in social activities and challenges in maintaining relationships with friends, family, and romantic partners [12]. Furthermore, UC is associated with decreased workforce participation, amplifying the economic burden of the disease [13].

The principal objective of UC treatment is to mitigate inflammation, thereby relieving symptoms and maintaining patients’ quality of life [14]. Patients with UC are typically treated with pharmacologic therapy, and multiple interventions are available. For patients experiencing mild-to-moderate symptoms, 5-aminosalicylic acid (5-ASA) is often the medication of choice [15]. However, whilst 5-ASA is associated with good clinical efficacy, its long-term effects and compliance are unknown, with adherence rates typically ranging between 40% and 50% [16]. Moreover, the use of 5-ASA can be associated with significant side effects, including hepatitis, pancreatitis, nephritis or nephrotoxicity, and pulmonary dysfunction. For those with moderate to severe UC, treatment usually involves the use of corticosteroids and immunosuppressive drugs [17]. However, the therapeutic benefits of this approach are often slow to manifest and also come with the risk of considerable side effects. These include an increased likelihood of developing non-Hodgkin’s lymphoma, allergic reactions, liver and pancreatic inflammation, infections, tuberculosis, and viral hepatitis [18].

UC is characterised by elevated levels of reactive oxygen species, produced by activated macrophages and neutrophils within the inflamed intestine [19], alongside oxidative stress imbalances [20] and a compromised intestinal antioxidant defence system [21]. There is a growing interest in alternative therapeutic approaches for UC management [22], with dietary interventions gaining attention for their potential to modify gut microbiota composition [23]. Natural antioxidative and anti-inflammatory agents have been proposed as promising alternatives to pharmaceutical treatments due to their potential to reduce gastrointestinal inflammation with fewer side effects, a hypothesis supported by recent clinical and animal studies [14,24]. Among these, anthocyanins, a prominent subclass of dietary flavonoids abundant in deeply pigmented fruits, exhibit potent antioxidant properties [25]. Studies indicate their benefits in mitigating heart disease, certain cancers, and inflammatory disorders [26], though research into their specific efficacy in UC remains in the early stages.

Animal studies have demonstrated that consuming anthocyanin-rich bilberries can significantly attenuate the severity of disease activity and lower serum levels of pro-inflammatory cytokines [27]. Building on these findings, two subsequent human clinical trials provided further evidence of the benefits of bilberry supplementation. The first study revealed that a 6-week regimen of bilberry supplementation led to a marked improvement in endoscopic disease activity and a significant reduction in faecal calprotectin levels [14]. The second showed that bilberry supplementation decreased levels of the pro-inflammatory cytokines IFN-γ and TNF-α in colon biopsy samples, as well as reduced serum levels of TNF-α and MCP-1 [26]. Notably, neither of these investigations incorporated a control group or assessed patient-reported outcomes. Montmorency tart cherries, known for their high anthocyanin content [28], have also shown promise in this area. Supplementation with Montmorency tart cherries has been found to increase the presence of Bacteroides in the gut [29], a bacterial genus known to reduce colon inflammation in IBD [30]. Given that biochemical analyses indicate Montmorency tart cherries have greater anthocyanin levels than most other dark fruits [28], there is a compelling rationale to explore whether their clinical efficacy might surpass the benefits previously observed with bilberry supplementation in patients with UC.

### 1.1. Rationale

To date, no placebo-controlled randomised trials have evaluated the efficacy of Montmorency tart cherry supplementation in patients with UC. However, our research indicates a strong willingness among this patient population to consider Montmorency tart cherry supplementation [31], reflecting substantial patient interest and engagement for a randomised clinical trial in this area. Consequently, further investigation into the potential health benefits of tart cherries for UC patients could hold significant practical and clinical importance.

### 1.2. Aims and Objectives

This trial aims to evaluate the effects of twice-daily Montmorency tart cherry supplementation on various health outcomes in patients with UC compared to placebo. The primary objective is to assess the impact of tart cherry supplementation on self-reported quality of life, measured using the Inflammatory Bowel Disease Quality of Life Questionnaire (IBDQ), relative to the placebo group. Secondary objectives include determining whether tart cherry supplementation affects additional health-related questionnaires and biological markers.

### 1.3. Hypotheses

We expect that Montmorency tart cherry supplementation will significantly enhance participants’ self-reported quality of life compared to placebo. Additionally, improvements in responses to other health-related questionnaires are anticipated, along with marked reductions in peripheral blood biomarkers indicative of lower inflammation. Furthermore, Montmorency tart cherry supplementation is projected to promote more stable and beneficial modulation of the gut microbiome relative to placebo.

## 2. Materials and Methods

### 2.1. Study Design and Setting

**Figure 1:**
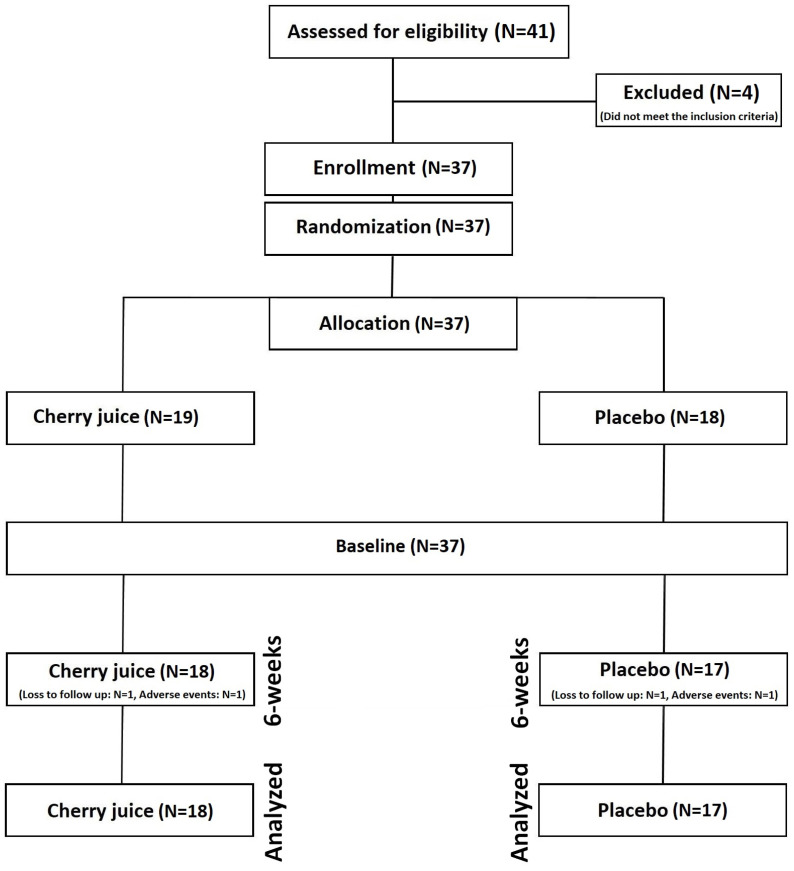
Consort diagram showing of participant flow throughout the study.

The comprehensive protocol for this study, detailing the study setting, CONSORT diagram, randomisation process, recruitment strategy, and sample size calculation, has been previously published [32]. The trial was conducted in accordance with the latest guidelines for reporting and conducting parallel-group randomised trials [33]. Data collection took place at the University of Hertfordshire in South England and the University of Central Lancashire in North West England. The trial employed a 6-week parallel-group design with randomised allocation and a placebo control (Figure 1). The trial duration was adopted in accordance with previous analyses exploring the effects of anthocyanin-rich fruit supplementation in patients with UC [14]. Eligible participants, following confirmation of suitability and enrolment, were randomly assigned using computer-based software (Random Allocation Software 2.0) to one of two groups for the 6-week intervention: (1) Montmorency tart cherry supplementation or (2) placebo. The random allocation was undertaken by the lead researcher and patients were assigned to their allocated trial arm during the data collection process. Primary and secondary outcomes were evaluated at baseline and post-intervention at 6 weeks, as outlined below. Consistent with prior research on UC management, the primary outcome was quality of life, measured using the IBDQ. Secondary outcomes included additional health-related questionnaires and biological markers.

### 2.2. Inclusion and Exclusion Criteria

#### 2.2.1. Inclusion Criteria

Eligibility criteria for this study required participants to meet the following conditions: (1) a confirmed diagnosis of UC for at least six months, (2) mild-to-moderate disease activity at enrolment, (3) an age range of 18 to 65 years, and (4) stable medication use for a minimum of three months. These inclusion criteria were established based on previous randomised trials involving UC patients [34,35]. Mild-to-moderate disease activity was defined as a Mayo Clinic Score between 3 and 10, consistent with the British Society of Gastroenterology consensus guidelines [36].

#### 2.2.2. Exclusion Criteria

Participants were excluded from the trial if they had diabetes, HIV, rheumatoid arthritis, or other autoimmune diseases, as well as Hepatitis B or C infections. Additional exclusion criteria included the presence of abscesses, unstable medical conditions that could interfere with study completion, or known food allergies to cherries.

### 2.3. Sample Size

To date, no studies have evaluated the efficacy of Montmorency tart cherry supplementation in patients with UC. Consequently, a pragmatic a priori sample size calculation was conducted based on a prior trial investigating the effects of bilberry supplementation on UC-related quality of life changes, measured using the Short Inflammatory Bowel Disease Questionnaire [14]. This calculation determined that a sample size of 32 participants would be required to achieve a statistical power of 80% (β = 0.80) at a 5% significance level (α = 0.05), allowing for the detection of a 6.5-point improvement in quality-of-life scores. Accounting for an anticipated 20% attrition rate, it was estimated that 20 participants per group, with a total of 40 participants, would be necessary.

### 2.4. Participants and Recruitment

Both males and females of diverse races and ethnicities who lived in Hertfordshire and Lancashire and their surrounding areas were recruited for this trial. Recruiting materials were placed via the local Crohn’s and Colitis UK (CCUK) network, the CCUK website, as well as using social media. Recruitment commenced in September of 2023 until July of 2024, with formal data collection ending in September of 2024. Individuals expressing interest in the study were encouraged to contact the research team for additional information and to address any questions regarding participation. Written informed consent was obtained from participants who agreed to enrol, and all participants were instructed to continue their routine medications and dietary habits throughout the study.

### 2.5. Ethical Approval and Trial Registration

This study was granted ethical approval by the University of Hertfordshire Health, Science, Engineering and Technology Ethics Committee (cLMS/SF/UH/05240; 20/07/2023) and the University of Central Lancashire HEALTH Ethics Committee (HEALTH 01104 CA Ext; 20/07/2023), and all participants submitted written informed consent before participating, adhering to the principles stated in the Declaration of Helsinki. The trial was preregistered on clinicaltrials.gov (NCT05486507).

### 2.6. Intervention

After completing their initial data collection, participants were provided with either Montmorency tart cherry concentrate or a placebo. Participants were blinded to their allocation. In accordance with previous trials examining the clinical efficacy of Montmorency tart cherries, participants were required to maintain their habitual diet and exercise routines throughout the intervention period, refrain from consuming any multivitamin or antioxidant supplements [37], and also asked to keep a 4-day diet diary prior to the baseline and 6-week data collection points [38,39,40,41]. This was to examine any differences in dietary patterns between groups and to explore whether participants made alterations to their nutritional approach that could influence the study outcomes. Diet diaries were analysed using WinDiets Nutritional Analysis Software Suite Version 1.0 (Robert Gordon University, Aberdeen, UK), allowing daily energy intake, fat, saturated fatty acids, protein, carbohydrate, sugars, fibre, alcohol, vitamin A, thiamine, riboflavin, niacin, vitamin B6, vitamin B12, folate, vitamin C, vitamin D, vitamin E, calcium, salt, iron, zinc, and selenium to be examined [42].

During the post-intervention data collection session, participants were instructed to return any unused supplementation, allowing for the calculation of actual supplement/placebo consumption and compliance rates (%). To estimate the average daily energy intake (kcal/day) and average daily sugar intake (g/day) from the supplementation/placebo, the consumed supplementation amount was multiplied by the energy and sugar content provided by the manufacturer and then incorporated into the information obtained from the diet diaries. To evaluate the efficacy of blinding, participants were asked at the end of their post-intervention session to indicate whether they believed they had been assigned to the supplement or placebo group. Additionally, loss to follow-up and adverse events were systematically recorded in both trial arms.

#### 2.6.1. Montmorency Tart Cherry

For a period of 6 weeks, participants assigned to this group consumed 60 mL of Montmorency tart cherry concentrate (Montmorency Tart Cherry Juice Concentrate, King Orchards, Central Lake, MI, USA) each day in two 30 mL servings [38,43,44]. The concentrate was diluted with 100 mL of water to make each 30 mL serving into a 130 mL beverage. Therefore, each day, 130 mL of the diluted tart cherry supplement was taken in two equal doses: in the morning and in the evening. A 30 mL dose of Montmorency tart cherry concentrate includes 80 Kcal, 19 g of carbohydrates, of which 15 g are sugars, 1.1 g of protein, and 1 g of fibre, according to the manufacturer. Previous phytochemical analyses have shown that 30 mL of Montmorency tart cherry concentrate contains 9.117 mg/mL of anthocyanins and 6.63 total phenolics/mL (expressed as gallic acid equivalents) [44,45].

#### 2.6.2. Placebo

The placebo was prepared in accordance with previous trials, that have demonstrated this form of placebo production to be an efficient blinding strategy [38,43]. For a total of 6 weeks, participants assigned to the placebo group ingested 60 mL each day [38,43]. The placebo was also diluted with 100 mL of water and taken twice a day in equal amounts, just like the supplement condition. Using a Professional series 750 blender (Vitamix, Olmsted Falls, OH, USA), the placebo was prepared by combining 100% unflavoured maltodextrin carbohydrates (MyProtein, Cheshire, UK) with drinking water. Maltodextrin was combined with water to make 1.0 litre of placebo concentrate, resulting in 20 g of maltodextrin per 30 mL dosing, matching the carbohydrate content of the Montmorency tart cherry supplement.

In order to replicate the colour of the Montmorency tart cherry concentrate, equal amounts of red and black food colouring were added. To achieve the desired flavour, 6 mL cherry flavouring (Special Ingredients, Yorkshire, UK) and 15 g citric acid (WholeFoods Earth, Kent, UK) were then added. A 30 mL dose of placebo concentrate includes 0 mg of anthocyanins and contains 80 Kcal, 20 g of carbohydrates, of which 0 g are sugars, 0 g protein, and 0 g fibre. To further promote effective blinding, identical opaque plastic bottles without any labels, were supplied to participants in both the placebo and Montmorency tart cherry trial arms, with the only difference being the solution that they contained.

### 2.7. Data Collection

All measurements were made inside either the Science Building at the University of Hertfordshire or the Nutrition Suite at the University of Central Lancashire and were undertaken in an identical manner across both locations and occasions, i.e., at baseline and 6 weeks.

#### 2.7.1. Demographic, Anthropometric and Health Information

Baseline data on participants’ ages, years since diagnosis, sex, race, smoking status, and medication use were collected through self-reported questionnaires. Anthropometric measurements included body mass (kg), height (m, measured without shoes), and body mass index (BMI, kg/m^2^). Height was measured using a stadiometer, and body mass was recorded using standard weighing scales. Peripheral systolic and diastolic blood pressure readings were obtained using a non-invasive, automated blood pressure monitor in accordance with the guidelines of the European Society of Hypertension [46]. Three measurements were taken, each spaced 1 min apart, with the mean of the last two readings used for analysis.

#### 2.7.2. Questionnaires

The primary outcome measure for this study was quality of life, assessed using the IBDQ. The IBDQ is a widely validated tool specifically developed for individuals with IBD [47,48]. It comprises 32 items scored on a 7-point Likert scale, with total scores ranging from 32 to 224, where higher scores reflect better IBD-related quality of life. A ≥16-point improvement in the total IBDQ score is considered clinically significant [49]. In addition to providing a total score, the IBDQ evaluates four specific dimensions: bowel symptoms, systemic symptoms, emotional health, and social function.

Secondary outcome measures included the Simple Clinical Colitis Activity Index (SCCAI), the IBD Fatigue Scale, the International Physical Activity Questionnaire-Short Form (IPAQ-SF), and the Hospital Anxiety and Depression Scale (HADS). The SCCAI is a validated symptom-based index comprising six items, with scores ranging from 0 to 19, where higher scores indicate more severe symptoms [50]. The IBD Fatigue Scale, a disease-specific instrument, evaluates fatigue in individuals with IBD [51]. It has two components: the first assesses fatigue levels using five questions (IBDF 1), and the second measures the impact of fatigue on quality of life using 30 questions (IBDF 2). Responses are scored on a 0–4 Likert scale, yielding a maximum score of 20 for IBDF 1 and 120 for IBDF 2, with higher scores reflecting greater fatigue and its impact. The IPAQ-SF is a validated nine-item questionnaire assessing physical activity across four categories: vigorous-intensity activity, moderate-intensity activity, walking, and sitting. This instrument allows for the calculation of weekly energy expenditure (Kcal) [52]. HADS is a validated 14-item scale used to evaluate symptoms of anxiety and depression. It consists of two subscales: one for depression (HADS-D) and one for anxiety (HADS-A), each containing seven questions. Scores for each subscale range from 0 to 21, with higher scores indicating greater levels of depression or anxiety [53].

#### 2.7.3. Blood Samples

Venous haematological samples were collected from the antecubital vein (12 mL) directly into uncoated blood collection tubes (Fisher Scientific, Hampton, NH, USA). Blood was allowed to clot for 30 min before serum separation by centrifugation at 2000× *g* for 10 min at 4 °C. Serum was aliquoted and stored at −20 degrees Celsius until assayed. A multiplex approach was adopted to develop a bespoke panel (Legend Plex, BioLegend Ltd., London, UK) to enable measurement of pro-inflammatory cytokines TNF-α, IL-1β, IL-6, IL-12p70 and CCL-2 (pg/mL) using an Agilent NovoCyte flow cytometer (Agilent Technologies, Cheshire, UK) according to the Legend Plex set up instructions.

#### 2.7.4. Faecal Samples

##### Faecal Calprotectin

To evaluate biological changes in intestinal inflammation, faecal samples were collected from participants in both groups at baseline and following the 6-week intervention. These samples were analysed for faecal calprotectin, which is considered the gold standard biomarker for assessing intestinal inflammation [54]. Faecal samples were provided by the participants prior to arriving on site for each visit. Faecal matter was prepared using the Calex Cap system with test cassettes according to manufacturer’s instructions (Buhlmann Group, Bremen, Germany). Samples were read using the Quantum Blue Reader III device (Buhlmann Group, Bremen, Germany).

##### Faecal Microbiota Communities

Ulcerative colitis is characterised by dysbiosis of the gut microbiota [55]. Indeed, individuals with ulcerative colitis have been demonstrated to exhibit diminished diversity of their gut microbiota [56]. Therefore, microbiota communities in the gut were also measured from faecal samples to determine changes in the communities. Specifically, alpha and beta diversity were measured to assess any changes in gut microbiota that might occur as a function of the intervention adopted in this trial. In accordance with current recommendations, alpha diversity was measured using the Shannon Diversity Index, whereas Beta diversity was quantified using the Bray–Curtis Dissimilarity [57].

Shotgun sequencing metagenomic methods were adopted, where sequencing was completed using an Oxford Nanopore minion MK1c sequencing device (Oxford Nanopore Technologies, Oxford, UK) with minKNOW software (v.23.04.8, Oxford Nanopore Technologies, Oxfordshire, UK). Using up to 100 mg of faecal matter for each sample, DNA was extracted using a pre-prep DNA/RNA shield (Zymo Research, Tustin, CA, USA) and then a high-molecular weight (HMW) Magbead DNA extraction kit (Zymo Research, Tustin, CA, USA). DNA was assessed for quantity and quality using Nanodrop (Thermo-Fisher Scientific, Waltham, MA, USA), Qubit (Agilent, Santa Clara, CA, USA), and Tapestation (Agilent, Santa Clara, CA, USA). A DNA Integrity Number (DIN) value of 6.0 was considered acceptable for sequencing.

The sequencing was carried out using Oxford Nanopore technologies R9 flow cell (FLO-MIN106) and SQK-LSK110 according to the original gDNA sequencing protocol without the fragmentation step (available at https://nanoporetech.com/document/genomic-dna-by-ligation-sqk-lsk110 accessed on 6 July 2024). For barcoding, the 96 Native barcoding expansion was used (EXP-NBD196). In brief, after the DNA end-prep was performed, 22.5 μL of approximately 500 ng of each sample was ligated with 2.5 μL of unique barcode and 25 μL of Blunt/TA Ligase Master mix from New England Biolabs (NEB, Ipswich, MA, USA) at room temperature for 10 min. Each sample was then mixed by hula mixer with 50 μL AMPure XP beads (Beckman Coulter, Buckinghamshire, UK) for 5 min before being washed 2x with 70% ethanol. Samples were eluted in 25 μL nuclease-free water and quantified on Qubit before being pooled together to make up the library for a target of 700 ng barcoded DNA. This gDNA library then underwent adapter ligation and cleanup as described in the SQK-LSK110 protocol. Additional reagents for ligation sequencing were obtained from New England Biolabs (NEB, Ipswich, MA, USA) as per Oxford Nanopore’s recommendations. Positive controls were performed using the nanopore kit EXP-CTL001 according to the manufacturer instructions. Negative controls were performed by running through DNA extraction protocol on nuclease-free water and run through the nanopore.

Sequencing was undertaken using MinKNOW software version 23.04.8, runs were allowed to go on for at least 24 h or when active pores reached <10. Base calling was undertaken using a Guppy base caller (v.6.5.7) set to a fast base calling protocol. All other settings on minION set up remained at default. Output was saved as FASTQ files with a minimum quality score of 7.

The Chan/Zuckerberg-ID (CZ-ID) online metagenomic next-generation sequencing (mNGS) nanopore pipeline was used to process the fastq files [58]. The fastq files were quality-filtered using fastp before human data were removed using minimap2. Data were subsampled to 1 million reads. Assembly was undertaken by metaFlye, and the contigs assembled with minimap2 and reads were compared with either the nucleotide (NT) or gene encoding (NR) databases from the National Center for Biotechnology Information (NCBI). The sample report was downloaded from CZ-ID and the normalised measure of bases per million was used to determine the relative abundance at the genus level (The CZ-ID pipeline is available at: https://chanzuckerberg.zendesk.com/hc/en-us/articles/13756558532884-CZ-ID-Pipeline-Overviews accessed on 6 July 2024). The data were then input into R (v.4.3.2) and the package vegan (v.2.8) was used to assemble data into an operational taxonomic units (OUT) table.

Phyloseq v.1.46.0 R package [59] was used to determine the abundance of bacteria at different taxa levels. Phylogenetic trees and taxonomy tables were obtained from the National Centre for Biotechnology Information (NCBI, available at https://www.ncbi.nlm.nih.gov/datasets/docs/v2/reference-docs/command-line/datasets/ accessed on 6 July 2024). Data were processed and trimmed to remove low-abundance taxa and analysed using Deseq2 v.1.42.1 R package [60]. The controls were analysed using Decontam v.1.22.0 R package [61]. Positive and negative controls were analysed using the prevalence algorithm to determine if certain genera were contaminants. Using this method, Staphylococcus was determined to be a contaminant and was removed from the analysis.

### 2.8. Statistical Analyses

Continuous experimental variables are expressed as means accompanied by their respective standard deviations, while categorical variables are reported as percentages (%) or frequencies (N). Comparisons of compliance levels (%) between trial arms were performed using a linear mixed-effects model, with trial arms included as a fixed factor and random intercepts assigned to participants. All intervention-related analyses adhered to an intention-to-treat framework. The effects of the intervention on all outcome measures were assessed by comparing the changes from baseline to 6 weeks between the two trial arms using linear mixed-effects models. These models included a trial arm as a fixed factor and random intercepts by participants [43]. For each model, the mean difference in change between groups (*b*) and the corresponding 95% confidence intervals (CIs) were reported, with analyses conducted using the restricted maximum likelihood method [43]. Effect sizes for changes from baseline to 6 weeks were calculated using Cohen’s d, as outlined by McGough and Faraone [62], where values of 0.2, 0.5, and 0.8 correspond to small, medium, and large effects, respectively [63].

Blinding efficacy was evaluated using a one-way chi-square (Χ^2^) goodness-of-fit test, while two-way Pearson chi-square tests of independence were applied for bivariate cross-tabulation analyses between trial arms. These analyses assessed the number of participants lost to follow-up and the incidence of adverse events in each group. Chi-square probabilities were derived using Monte Carlo simulation. The aforementioned analyses were performed using SPSS v29 (IBM Inc., SPSS, Chicago, IL, USA).

Data obtained as a function of the faecal microbiota communities were analysed using R software (v.4.3.2). Differences in alpha diversity using the Shannon diversity index were compared between cohorts using a Kruskal–Wallis test in accordance with [57]. Weighted Bray–Curtis dissimilarity was undertaken in community structure between trial arms using PERMANOVA to calculate *p*-values [57], with principal coordinate analysis plots (PCoA) used to visualise beta diversity [64]. All statistical analyses were performed with statistical significance considered at the *p* ≤ 0.05 level.

## 3. Results

### 3.1. Baseline Demographic, Anthropometric, and Health Information

Characteristics of participants are presented in Table 1.

### 3.2. Compliance, Loss to Follow Up and Adverse Events

Our initial sample size calculation required thirty-two participants, with an additional 8 to account for an expected 20% dropout rate, with a total N of forty. We enrolled a total of thirty-seven participants, and with only two total patient dropouts, our trial remained appropriately powered, as the actual non-completion rate was lower than anticipated. Whilst all 35 participants who completed the trial provided data for the primary outcome and other associated measures, challenges arose with the collection of blood and faecal samples. Due to difficulties in obtaining venous blood samples in this population and participant sensitivity to provide faecal samples, 27 participants completed blood sampling at both baseline and 6 weeks, and only 26 participants provided faecal samples at these time points.

Total trial completion numbers in each group were tart cherry N = 18 and placebo N = 17, and the numbers of adverse effects were cherry N = 1 and placebo N = 1, with both being a flare-up of UC symptoms (Figure 1). The chi-square tests were non-significant (*p* = 0.969), indicating that there were no statistically significant differences between trial arms in either loss to follow-up or adverse events. There was no statistically significant difference (*p* = 0.531) in compliance between the tart cherry (95.8%) or placebo (98.3%) trial arms.

### 3.3. Blinding Efficacy

Of the 35 participants that completed the trial, N = 16 correctly identified their designated trial arm, and the chi-squared test was non-significant (*p* = 0.612), indicating that an effective blinding strategy was adopted.

### 3.4. Diet Diaries

Increases in daily sugars were significantly greater in the tart cherry arm compared to placebo (Table 2). No other significant differences (*p* > 0.05) were found.

### 3.5. Questionnaires

In relation to the primary trial outcome, improvements in total IBDQ scores in the tart cherry trial arm are significantly greater than in the placebo (Table 3). Furthermore, the results also show that improvements in the constituent components of the IDBQ scale of bowel symptoms, IBDQ emotional health, and IBDQ social function as well as the SCCAI were significantly greater in the tart cherry trial arm compared to the placebo (Table 3). No other significant differences (*p* > 0.05) were found.

### 3.6. Blood Samples

No significant differences (*p* > 0.05) in inflammatory biomarkers were found (Table 4).

### 3.7. Faecal Samples

#### 3.7.1. Faecal Calprotectin

Reductions in faecal calprotectin in the tart cherry trial arm were significantly greater than in the placebo (Table 5).

#### 3.7.2. Faecal Microbiota Communities

**Figure 2:**
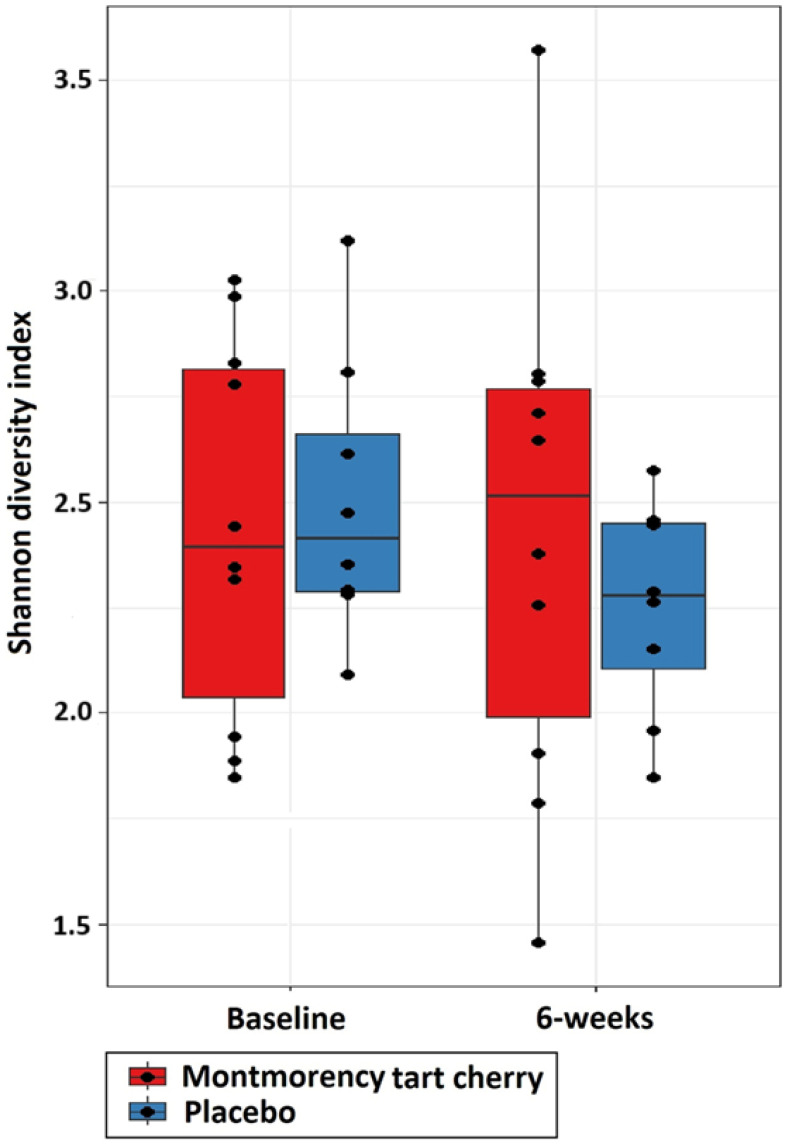
Comparison of Shannon diversity index between placebo and tart cherry groups at baseline and 6 weeks.

**Figure 3:**
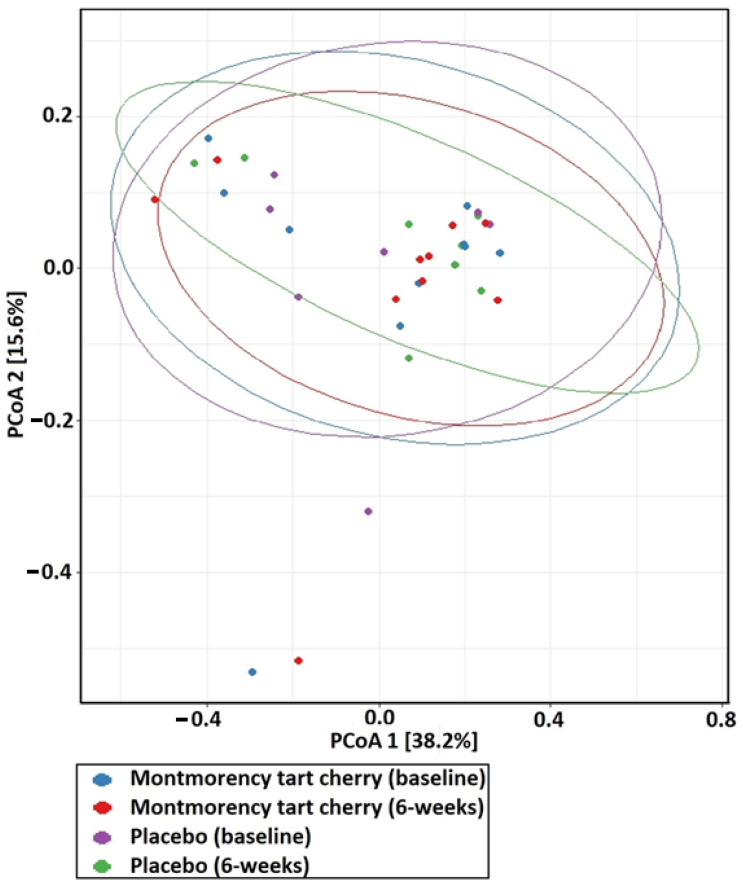
Beta diversity plotted using PCoA based on the Bray–Curtis dissimilarity between trial arms and study timepoints.

No significant differences (*p* = 0.460) in alpha diversity were observed (Figure 2). Similarly, no significant differences (*p* = 0.318) in beta diversity were shown (Figure 3).

## 4. Discussion

This trial aimed to evaluate the effects of a 6-week regimen of twice-daily Montmorency tart cherry supplementation on various health indicators in patients with UC relative to placebo. Notably, this study represents the first randomised controlled trial employing a parallel placebo-controlled design to investigate the impact of Montmorency tart cherry supplementation in this patient population. The primary objective was to examine the influence of tart cherry supplementation on self-reported quality of life via the IBDQ compared to placebo. Secondary objectives included assessing its effects on additional health-related outcomes and biological indices.

In relation to the primary outcome, our hypothesis was supported as the tart cherry trial arm demonstrated significantly greater improvements in IBDQ scores compared to placebo. Moreover, the observed improvement importantly exceeded the threshold for clinically meaningful change [49]. Furthermore, the results also showed that the constituent components of the IDBQ scale of bowel symptoms, IBDQ emotional health, and IBDQ social function as well as the SCCAI were significantly greater in the tart cherry trial arm compared to placebo. UC is known to have a profoundly negative effect on patients’ health perception, self-image, and social participation, as well as diminishing overall health-related quality of life [10,12,13]. Further, along with the clinical manifestations of UC, this condition also imposes a significant fiscal burden on global healthcare systems [4,6,7]. Consequently, the observations from this trial may have considerable clinical relevance. Whilst pharmaceutical interventions for UC have been shown to exhibit good efficacy, they are associated with significant side effects [18], significant monetary restrictions on healthcare budgets, and low adherence rates [16]. Therefore, the findings from the current trial lend support to the concept that twice-daily tart cherry supplementation can improve IBD-related quality of life as well as the severity of symptoms and therefore may be important in the management of UC.

Although no differences in blood inflammatory biomarkers or faecal microbiota communities were evident, this trial, in addition to demonstrating improved self-reported clinical outcomes via the IBDQ and SCCAI, revealed that reductions in faecal calprotectin were significantly greater in the tart cherry trial arm compared to placebo. Faecal calprotectin is an important biomarker used to assess disease severity and response to treatment in the evaluation of inflammatory bowel disease [65]. In UC, elevated calprotectin is demonstrative of augmented intestinal inflammation [66]. Calprotectin is also recognised as an effective predictor of histologic healing after treatment for active UC [67], an outcome associated with improved clinical outcomes, including a reduced risk of clinical relapse, surgery, and fibrosis [68].

Importantly, it appears that Montmorency tart cherry supplementation can mediate reductions in intestinal inflammation and promote histologic healing, effects which were likely responsible for the improvement in clinical outcomes in this trial arm. These benefits may also be attributed to gut microbiota-derived metabolites produced in response to tart cherry supplementation [69]. Polyphenols in tart cherries are metabolised by gut bacteria into bioactive compounds with anti-inflammatory and antioxidant properties, which may explain the reductions in calprotectin and improvements in symptoms. However, observing differences in microbiota diversity could require a supplementation phase longer than 6 weeks [40]. Previous research has indicated that while metabolic changes can occur relatively quickly, alterations in microbial diversity can necessitate longer intervention periods to become apparent [40]. 

It is important to also acknowledge that despite the important improvements in the IBDQ and SCCAI in the tart charry trial arm, the dietary analyses showed that this supplement was associated with statistical increases in sugar intake in relation to placebo. It is apparent that this observation was caused by the increased sugar content in the tart cherry supplement and, although not explored as part of this trial, Chai et al. [70] showed that tart cherry supplementation significantly increased plasma glucose levels. Previous observations have demonstrated a mechanistic connection(s) between IBD and diabetes [71,72,73], associations between sugar intake and IBD risk [74], and also importantly between sugar intake and exacerbation of symptoms [75]. However, previous trials have also demonstrated that tart cherry supplementation can mediate significant improvements in glucose control and haemoglobin A1c, markers pertinent to diabetes aetiology [76,77]. Therefore, it is important to examine the longer-term effects of tart cherry supplementation in UC patients.

Overall, the current trial demonstrated a successful blinding strategy, as well as a very low number of adverse events, a good level of compliance, and a high retention rate in the tart cherry group. Therefore, it can be concluded that tart cherry is a safe and tolerable modality in UC patients that can be incorporated easily into habitual dietary patterns. These observations, allied to the significant improvements in IBDQ and SCCAI indices in the tart cherry trial arm indicate that this supplement appears to represent an effective means to enhance health-related quality of life in UC patients. Owing to the statistically greater mean sugar and daily additional kilocalorie intake, in agreement with Kimble et al. [39] and Sinclair et al. [43], those seeking to utilise tart cherry juice as a dietary supplement on a long-term basis should seek to modify their daily dietary intake to account for the increase in daily sugars and Kcal.

As with any randomised controlled trial, this investigation is not without limitations. One potential drawback is that participants were randomised into their designated trial arms using a 1:1 allocation procedure without consideration for their current medical therapy. This lack of stratification may have influenced the variability in responses to tart cherry supplementation. Therefore, future studies should consider adopting a stratified random sampling approach based on patients’ pharmaceutical management at baseline to better evaluate the effects of tart cherry supplementation in the context of varying treatment regimens. Another limitation is the reliance on self-reported disease activity for patient eligibility, as opposed to clinician-verified assessments. Although the Mayo Clinic Scale, a widely recognised tool, was used to assess disease severity, it is inherently subjective and may lead to variability in patient classification due to the potential for over- or underestimation of symptoms. Future investigations should incorporate objective clinical assessments, such as histological or endoscopic evaluations, to provide a more robust and accurate measure of disease activity and therapeutic response. Additionally, while our findings suggest the potential benefits of tart cherry supplementation, these effects may not be uniform across all individuals with ulcerative colitis. The heterogeneity of the UC patient population means that certain subgroups may respond more favourably than others. To better understand which patient groups may derive the greatest benefit, future research should conduct stratified analyses based on factors such as disease severity, duration, medication use, and other relevant clinical characteristics.

## 5. Conclusions

The current placebo randomised controlled trial aimed to investigate the influence of 6 weeks twice daily Montmorency tart cherry supplementation, on various health indicators in patients with UC, compared to the placebo. The current trial supported our primary hypothesis that ingestion of twice daily tart cherry supplementation was able to mediate improved IBD-related quality of life compared to placebo and also secondary predictions concerning the severity of symptoms measured via the SCCAI. Given the profoundly negative effects of UC health-related quality of life and its fiscal implications for global healthcare systems, the findings from this trial indicate that twice-daily tart cherry supplementation can improve IBD-related quality of life as well as the severity of symptoms and therefore may be important in the management of UC.

## Figures and Tables

**Table 1:** Participant characteristics.

	Total		Montmorency Tart Cherry		Placebo	
	Mean	*SD*	Mean	*SD*	Mean	*SD*
Sex	Male = 40.0%		Male = 33.3%		Male = 47.1%	
	Female = 60.0%		Female = 66.7%		Female = 52.9%	
Age (yrs)	43.39	11.38	43.35	8.69	43.43	14.35
Mass (kg)	80.16	20.12	77.63	18.50	82.86	22.05
Stature (cm)	171.81	8.63	169.17	8.11	174.63	8.53
BMI (kg/m^2^)	27.02	6.03	26.95	5.12	27.10	7.06
Systolic blood pressure (mmHg)	125.29	13.42	127.26	13.97	123.20	12.95
Diastolic blood pressure (mmHg)	86.06	8.31	87.69	9.54	84.33	6.65
Smoking status	Yes = 6.4%		Yes = 0%		Yes = 14.3%	
	No = 87.2%		No = 88.2%		No = 85.7%	
	Previous = 6.4%		Previous = 11.8%		Previous = 0%	
Years since diagnosis	9.08	6.04	9.69	6.23	8.43	5.97
Ethnicity (%)	Caucasian = 90.4% Pakistani = 3.2% Arab = 3.2% African = 3.2%		Caucasian = 87.4% Pakistani = 6.3% Arab = 6.3%		Caucasian = 93.3% African = 6.7%	
Medication (%)	Balsalazide	10.0	Balsalazide	13.3	Balsalazide	6.7
	Mesalazine	36.7	Mesalazine	46.7	Mesalazine	26.7
	Lactoflorene	3.3	Lactoflorene	0.0	Lactoflorene	6.7
	Mesalazine suppository	6.7	Mesalazine suppository	13.3	Mesalazine suppository	0.0
	Mercaptopurine	6.7	Mercaptopurine	0.0	Mercaptopurine	13.3
	Allopurinol	6.7	Allopurinol	6.7	Allopurinol	6.7
	Infliamab	6.7	Infliamab	0.0	Infliamab	13.3
	Azathioprine	23.3	Azathioprine	20.0	Azathioprine	26.7
	Medical cannabis	3.3	Medical cannabis	0.0	Medical cannabis	6.7
	Infliximab	3.3	Infliximab	6.7	Infliximab	0.0
	Tofacitinib	3.3	Tofacitinib	0.0	Tofacitinib	6.7
	Adalimumab	3.3	Adalimumab	6.7	Adalimumab	0.0
	Vedolizumab	3.3	Vedolizumab	0.0	Vedolizumab	6.7
	Methotrexate	3.3	Methotrexate	0.0	Methotrexate	6.7

**Table 2:** Mean and standard deviation (SD) daily dietary outcomes for each trial arm.

	Montmorency Tart Cherry				Placebo				*b*	95% CI		*p*-Value	*d*
	Baseline		6-Weeks		Baseline		6-Weeks						
	Mean	*SD*	Mean	*SD*	Mean	*SD*	Mean	*SD*		Lower	Upper		
Energy intake (Kcal)	1790.00	429.98	1924.17	479.10	1793.42	471.43	2046.91	613.27	−119.33	−537.99	299.34	0.562	−0.23
Fat (g)	70.61	25.58	73.28	25.62	66.23	19.40	76.97	24.43	−8.06	−30.95	14.82	0.474	−0.29
Saturated fatty acids (g)	25.18	9.53	31.18	12.95	23.81	10.02	25.43	12.48	4.38	−7.27	16.03	0.446	0.31
Protein (g)	83.99	37.02	74.67	19.47	64.65	23.48	78.59	26.31	−23.26	−47.69	1.18	0.061	−0.77
Carbohydrate (g)	209.50	87.14	246.82	91.57	230.88	78.42	269.67	98.03	−1.47	−75.79	72.84	0.968	−0.02
Sugars (g)	72.86	45.78	95.19	40.43	118.94	63.43	94.44	70.73	***46.83***	***5.77***	***87.89***	***0.027***	***0.93***
Fibre (g)	14.96	7.22	16.25	6.68	16.23	6.30	17.60	8.18	−0.08	−6.04	5.88	0.978	−0.01
Alcohol (mL)	6.66	13.75	4.71	12.29	10.43	25.24	4.51	14.82	3.97	−6.28	14.21	0.432	0.31
Vitamin A (ug)	373.57	176.83	695.24	858.86	587.83	644.35	750.33	547.57	−159.17	−620.30	301.95	0.483	−0.28
Thiamine (mg)	1.49	0.88	1.11	0.42	1.35	0.65	1.59	0.80	−0.62	−1.31	0.07	0.075	−0.73
Riboflavin (mg)	1.67	0.64	1.06	0.44	1.47	0.59	1.64	1.55	−0.78	−1.61	0.06	0.067	−0.75
Niacin (mg)	38.30	22.19	29.71	11.27	27.50	13.46	37.22	15.77	−14.72	−32.71	3.28	0.104	−0.66
Vitamin B6 (mg)	1.33	0.68	1.04	0.31	1.39	0.80	1.62	0.59	−0.52	−1.09	0.05	0.073	−0.74
Vitamin B12 (mg)	4.55	2.32	3.04	1.98	4.09	2.91	5.73	6.81	−3.16	−6.57	0.25	0.068	−0.75
Folate (ug)	224.00	118.50	195.95	130.79	198.00	103.75	255.08	158.35	−85.13	−191.27	21.01	0.111	−0.65
Vitamin C (mg)	73.64	113.37	69.51	83.96	111.38	214.47	79.51	80.41	27.75	−103.47	158.96	0.666	0.17
Vitamin D (mg)	3.01	3.58	3.10	5.38	2.12	2.78	3.37	3.81	−1.16	−5.65	3.34	0.600	−0.21
Vitamin E (mg)	7.84	3.45	6.92	3.30	7.22	3.01	7.78	3.57	−1.48	−4.84	1.87	0.370	−0.36
Calcium (mg)	815.64	298.30	767.88	320.57	727.50	289.01	911.92	633.29	−232.17	−571.23	106.88	0.170	−0.56
Salt (g)	5.26	2.80	4.75	2.18	4.67	1.25	4.74	2.27	−0.58	−2.51	1.35	0.542	−0.24
Iron (mg)	10.41	4.96	8.70	3.70	9.62	1.77	11.35	5.32	−3.44	−7.53	0.65	0.095	−0.68
Zinc (mg)	9.44	5.38	7.63	2.68	7.42	2.51	9.46	4.44	−3.84	−8.00	0.31	0.068	−0.75
Selenium (mg)	48.14	26.05	43.74	22.42	50.00	25.61	60.83	34.86	−15.24	−42.69	12.22	0.263	−0.45

Notes: bold text = significant difference in the changes from baseline to 6 weeks between the two groups.

**Table 3:** Mean and standard deviation (SD) questionnaire outcomes for each trial arm.

	Montmorency Tart Cherry				Placebo				*b*	95% CI		*p*-Value	*d*
	Baseline		6-Weeks		Baseline		6-Weeks						
	Mean	*SD*	Mean	*SD*	Mean	*SD*	Mean	*SD*		Lower	Upper		
IBDQ Total	159.56	33.44	179.94	25.65	171.24	22.20	167.76	33.28	***23.86***	***6.63***	***41.05***	***0.008***	***0.95***
IBDQ Bowel systems	52.56	11.77	57.56	8.29	56.41	8.28	54.06	11.09	***7.35***	***1.39***	***13.37***	***0.017***	***0.85***
IBDQ Emotional health	55.00	12.64	64.89	11.33	61.35	8.84	61.65	12.48	***9.59***	***2.73***	***16.43***	***0.008***	***0.96***
IBDQ Systemic systems	22.67	5.73	25.72	6.09	22.41	5.30	22.88	6.56	2.58	−1.03	6.17	0.156	0.49
IBDQ Social Function	29.39	6.02	31.78	4.58	31.06	3.72	29.18	5.96	***4.27***	***0.69***	***7.91***	***0.021***	***0.82***
IBD-F 1	8.94	4.98	6.83	4.73	7.47	4.85	7.41	4.91	−2.05	−4.87	0.77	0.148	−0.50
IBD-F 2	33.06	22.37	21.72	21.70	23.88	20.81	20.71	18.37	−8.16	−20.65	4.34	0.193	−0.45
Simple Clinical Colitis Activity Index	5.22	2.90	2.89	2.30	3.76	2.11	5.47	5.06	***−4.04***	***−6.73***	***−1.35***	***0.004***	***−1.03***
HADS A	8.20	2.88	6.93	4.25	6.54	3.50	6.46	3.38	−1.19	−4.07	1.69	0.404	0.19
HADS D	5.00	3.30	4.27	2.91	5.69	3.45	4.69	2.59	0.27	−2.26	2.79	0.830	−0.32
IPAQ (Kcal)	4687.92	5100.07	5590.14	5598.00	3166.31	2752.68	3421.19	2587.35	647.34	−1702.70	2997.38	0.579	0.08

Notes: bold text = significant difference in the changes from baseline to 6 weeks between the two groups.

**Table 4:** Mean and standard deviation (SD) blood sample outcomes for each trial arm.

	Montmorency Tart Cherry				Placebo				*b*	95% CI		*p*-Value	*d*
	Baseline		6-Weeks		Baseline		6-Weeks						
	Mean	*SD*	Mean	*SD*	Mean	*SD*	Mean	*SD*		Lower	Upper		
IL-1β (pg/mL)	244.78	99.34	223.50	69.55	233.91	75.14	229.97	76.61	−17.35	−53.02	18.32	0.324	−0.41
TNF-α (pg/mL)	120.03	92.56	80.80	79.30	105.53	90.39	109.79	92.06	−43.50	−96.68	9.68	0.104	−0.70
CCL-2 (pg/mL)	268.12	147.05	338.54	103.57	343.54	167.75	331.14	193.02	82.84	−40.99	206.67	0.179	0.57
IL-6 (pg/mL)	24.54	20.93	16.96	12.96	38.20	31.38	35.53	28.54	−4.91	−19.78	9.96	0.500	−0.28
IL-12p70 (pg/mL)	79.37	35.01	77.07	31.67	102.79	40.67	105.61	49.44	−5.12	−23.33	13.10	0.566	−0.24

**Table 5:** Mean and standard deviation (SD) faecal calprotectin outcomes for each trial arm.

	Montmorency Tart Cherry				Placebo				*b*	95% CI		*p*-Value	*d*
	Baseline		6-Weeks		Baseline		6-Weeks						
	Mean	*SD*	Mean	*SD*	Mean	*SD*	Mean	*SD*		Lower	Upper		
Faecal calprotectin (µg/g)	251.65	262.14	151.81	228.23	55.36	60.85	81.71	76.69	***−136.17***	***−268.06***	***−4.28***	***0.044***	***−1.14***

## Data Availability

Data available on request.
